# Drug vaping applied to cannabis: Is “Cannavaping” a therapeutic alternative to marijuana?

**DOI:** 10.1038/srep25599

**Published:** 2016-05-26

**Authors:** Vincent Varlet, Nicolas Concha-Lozano, Aurélie Berthet, Grégory Plateel, Bernard Favrat, Mariangela De Cesare, Estelle Lauer, Marc Augsburger, Aurélien Thomas, Christian Giroud

**Affiliations:** 1 Forensic Toxicology and Chemistry Unit, University Centre of Legal Medicine, Geneva-Lausanne, Switzerland; 2 Institute for Work and Health (IST), University of Lausanne, University of Geneva, Lausanne, Switzerland; 3 Psychology and Traffic Medicine Unit, University Centre of Legal Medicine, Lausanne, Switzerland; 4 Department of Ambulatory Care and Community Medicine, University of Lausanne, Switzerland; 5 Faculty of Biology and Medicine, Lausanne University Hospital, University of Lausanne, Lausanne, Switzerland.

## Abstract

Therapeutic cannabis administration is increasingly used in Western countries due to its positive role in several pathologies. Dronabinol or tetrahydrocannabinol (THC) pills, ethanolic cannabis tinctures, oromucosal sprays or table vaporizing devices are available but other cannabinoids forms can be used. Inspired by the illegal practice of dabbing of butane hashish oil (BHO), cannabinoids from cannabis were extracted with butane gas, and the resulting concentrate (BHO) was atomized with specific vaporizing devices. The efficiency of “cannavaping,” defined as the “vaping” of liquid refills for e-cigarettes enriched with cannabinoids, including BHO, was studied as an alternative route of administration for therapeutic cannabinoids. The results showed that illegal cannavaping would be subjected to marginal development due to the poor solubility of BHO in commercial liquid refills (especially those with high glycerin content). This prevents the manufacture of liquid refills with high BHO concentrations adopted by most recreational users of cannabis to feel the psychoactive effects more rapidly and extensively. Conversely, “therapeutic cannavaping” could be an efficient route for cannabinoids administration because less concentrated cannabinoids-enriched liquid refills are required. However, the electronic device marketed for therapeutic cannavaping should be carefully designed to minimize potential overheating and contaminant generation.

The creativity of cannabis users leads to new methods of consumption. Although developments such as vaporizers, edible and liquid tinctures have become very popular alternatives to the traditionally smoked flower buds of cannabis, they show flaws in concentrations, contamination or efficiency over the day according to galenic forms^1^. Recently, consumption methods such as butane hashish oil (BHO, cannabis concentrate extracted with butane gas) called “dabbing”, have been increasingly observed^2^,^3^,^4^, especially on Web fora. By eliminating tobacco and increasing THC levels, these optimizations appear to be interesting improvements for the therapeutic use and delivery of cannabis. However, public health concerns remain because of the ease of preparation, more rapid THC delivery (i.e., a potentially higher risk of dependence) and overdose^5^,^6^.

Dabbing involves vaporizing cannabis concentrates (BHO or cannabis dabs) at 300–400 °C on a hot surface and then inhaling the vapor through a specialized pipe^7^,^8^. BHO is a viscous, sticky to hard, wax-like concentrate that contains mainly acid cannabinoids and terpenoids. After thermal treatment, inactive tetrahydrocannabinolic acid (THC-A) is decarboxylated into psychoactive tetrahydrocannabinol (THC). BHO is highly enriched in THC, and its concentration is typically 15 to 30 times higher than that found in flower buds^4^. However, the literature is very scarce concerning BHO preparation and composition. Commonly, a dab of the dense oil is placed on the end of a glass or titanium rod that has been heated. If the dab has been previously decarboxylated, the flame from a lighter is sufficient to vaporize the BHO, but if the dab has not been decarboxylated, a blowtorch is used to maximize the decarboxylation of THC-A into its psychoactive form, THC, and to vaporize the BHO concentrate^1^.

Nevertheless, the enormous growth and development of electronic cigarettes (or e-cigarettes) have enabled the development of electronic devices exclusively dedicated to direct dripping (the liquid is dripped onto the bridge of the atomizer, instead of relying on the cartridge filler)^9^ and dabbing. These devices are currently available on the Internet and cannabis derivatives shops. Furthermore, a profusion of recipes is available on the Internet to prepare liquid refills enriched with cannabinoids. In this regard, BHO can be dissolved in the commercial liquids used to refill e-cigarettes, constituting an alternative to direct BHO dabbing. With the several thousand flavored liquids commercially available, flavors added to liquid refills can mask the overwhelming typical odor of cannabis and can make its vaping more discreet^10^. Indeed, concentrating cannabinoids implies a concentration of terpenoids and odorant compounds, which renders dabbing an easily detectable method of cannabis consumption for people exposed passively to cannavaping. Other recipes mention mixtures of solvents and flavors elaborated according to home-made protocols, including the dissolving of BHO in various propylene glycol/glycerin mixtures^1^, sometimes with the addition of edible or aromatic oils such as coconut or cinnamon. This new method of cannabis consumption could be called “cannavaping”. Thus, “cannavaping” and BHO dabbing could be considered major risks of electronic device misuse mainly because of the uncontrolled cannabinoids compositions of liquid refills and dabs. Nonetheless, “cannavaping” (of liquid refills) with controlled cannabinoids compositions and concentrations offers a new opportunity to deliver therapeutic cannabis without the requirement of smoking. The regulations of European countries have recently registered positive concern with the therapeutic use of cannabinoids^11^. By November 2014, a total of 23 states in the U.S. had enacted laws legalizing medicinal use^3^.

Nevertheless, the possibility of e-cigarette multiuse for cannavaping has arisen, whereas the carbonyls and volatile toxic compounds in the vapors of conventional e-cigarettes are still discussed^12^,^13^,^14^,^15^. The multiple designs and settings at users’ disposal could lead to significant toxic compound levels even if it requires high settings, causing burnt off-flavors generally not appreciated by usual vapers, defined as vaping devices users^16^,^17^,^18^,^19^,^20^. Indeed, to increase the decarboxylation rate of inactive cannabinoids acids (in case of misuse), cannavapers need to use high power settings (by using high voltage and/or low ohmic resistance atomizers). Regular cannabis smokers will prefer e-cigarette aerosols with strong organoleptic qualities. Moreover, the unpleasant flavors of liquid refills heated at such high settings could be hidden by the strong flavor of cannabis terpenoids. Consequently, the investigations of e-cigarettes vapors should always consider carbonyls and volatile compounds monitoring. Although concerns about the generation of contaminants from glycerin and propylene glycol are well identified, other concerns that have received little attention until now include the potential for toxic effects from inhaled flavorings^21^.

The goal of this study was to evaluate the efficiency of cannabis vaping using commercially available e-cigarettes as an alternative to marijuana smoking for therapeutic cannabis delivery. The ease of manufacture of THC-enriched liquid refills for e-cigarettes (and its misuses), the possibility of generating THC-enriched aerosols at sub-pyrolysis temperatures, and the formation of potentially toxic by-products, i.e., volatile organic compounds (VOCs) and carbonyls residues were considered in this evaluation.

## Results

### Aerosol generation

The electrical settings of the e-cigarette and the choice of the value of the resistance are known to affect the amount and composition of the aerosol. The best compromise (optimum power) must be found between the value of the resistance and the battery voltage to generate the greatest amount of aerosol and THC and fewer toxic by-products. Overly high power can damage the coil and equipment and promote the formation of toxic compounds.

The current measured through the coil was 1.3 amperes (A) with a maximum variation of 0.1 A (7.7%) for all of the monitored puffs. This value complied with Ohm’s law because the battery provided 5 V, and the coil resistance was 3.8 Ω. The stability of the current intensity indicated that the batteries could provide fairly constant 6.4 W power output throughout the 20 puffs.

**Figure 1:**
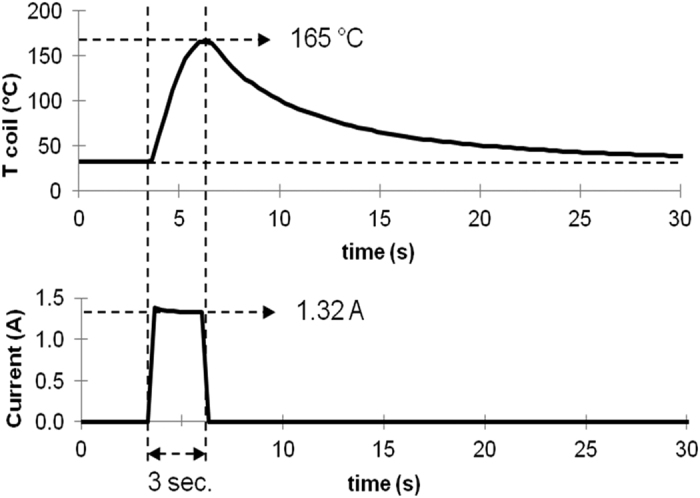
Temperature of the liquid near the coil and current monitored during one typical puff of 3 seconds.

The peak temperature of puffs measured in the close vicinity of the coil reached 165 °C on average, with a standard deviation of 16 °C for all of the monitored puffs. This value could have been underestimated because the sensor was not in direct contact with the metallic coil. Nevertheless, this recorded temperature was probably characteristic for the few milligrams of liquid atomized in one puff. It could be observed that the temperature increased until the current was turned off; therefore, the temperature could be greater given a longer puff (Fig. 1). However, at this level of temperature, power and puff duration, the smoke generation met the “dry puff conditions” defined in a previous study^22^. “Dry puffs” occur when there is an insufficient supply of liquid to the wick, causing high temperature generation and liquid overheating, resulting in the development of a strong unpleasant taste. During the vaping cycle of 20 minutes, the temperature of the clearomizer remained relatively stable with an increase of less than 3 °C because the e-cigarette had sufficient time to cool at room temperature before the subsequent ignition.

### Cannabinoids standards mixed in liquid refills

The decarboxylation yield of THC-A was obtained by comparison of cannabinoids aerosol profiles generated from liquid refills containing either pure THC (Design A) or a mixture of THC-A and THC (Design B) (Table 1). After subtracting the THC level initially present in the liquid, we found that a THC-A concentration of 81 mg/g of liquid led to a THC concentration in the condensed vapor of 5.2 mg/g of liquid consumed, resulting in a conversion rate of only 8.4%. Therefore, the decarboxylation of THC-A with the type of e-cigarette used in our study was possible, albeit with poor performance. The temperature monitored during puff cycles showed an average maximum of 165 °C, which was quite sufficient to promote full decarboxylation. The short duration of each puff (3 seconds) could appear therefore to be the main limiting factor because the best combination of temperature and heating time is required for optimal decarboxylation. During the 3 sec puffs, the temperature exhibits a gradual elevation from low levels up to the maximum of 165 °C measured (Fig. 1). Thus, although the final temperature is sufficient for conversion, this refers only to the peak temperature which is probably reached at the end of the puff. However, if longer puffs could improve the decarboxylation rate of THC-A, they could also generate toxic contaminants (mainly carbonyls) and cause an unpleasant taste of the aerosol^23^.

The total recovery yield of THC in the mainstream smoke of the cannabis cigarette (Design C) reached 5.8% because only 3.8 mg of THC were recovered in the smoke trapped in the cartridges from an initial amount of 65 mg of THC-A. In contrast with e-cigarette vaping, a large amount of sidestream smoke was also formed and released into the exposure room. However, the small amount of THC found in the mainstream smoke conformed with the THC concentrations found in the literature for cannabis cigarettes and with the daily dosage recommended for therapeutic cannabis administration: oral dronabinol - between 2.5 and 90 mg^24^; vaporization of liquid THC deposited on a pad - between 2 and 8 mg^25^,^26^; and vaporization of cannabis (200 mg with THC percentage of 4.2%) - final THC amount: 8.2 mg^27^. Nevertheless, the THC concentration found in the mainstream smoke in our study was at least three times less than the THC concentration recommended for vaporization of therapeutic cannabis in the Netherlands (200 mg of cannabis with a THC percentage between 6 and 19%), with theoretical concentrations between 12 and 38 mg^28^.

As a result, potentially to feel the same effects as with a cannabis cigarette delivering 3.8 mg of THC, the enormous quantity of 720 mg of liquid refill (spiked at the same cannabinoids concentration as in cannabis) should be vaped. These data showed that vaping the desired concentrations of cannabinoids could be only achieved using concentrates such as decarboxylated BHO solutions (as described in this work).

### Cannabinoids content in mixtures of refill liquids fortified with BHO and vapors from these liquid refills (commercial liquid and pure propylene glycol PG)

#### Decarboxylation of THC-A and BHO solubilization in liquid refills

Crude unheated BHO, prepared according to the procedure briefly described below, contained 73 ± 2.5% (w/w) of THC-A and less than 3 ± 0.2% (w/w) of THC. The raw decarboxylated BHO contained 59 ± 1.2% (w/w) of THC. As shown in Table 1, the decarboxylation step (2 hours, 120 °C) was complete because only traces of THC-A could be detected after thermal processing. The solubilization of crude, not unheated, BHO was difficult because of the presence of insoluble matter and fine plant fragments and impurities. Indeed, BHO concentrates are strongly lipophilic, while liquids refills are very hydrophilic, and both are highly viscous. Vortex-mixing of the decarboxylated BHO resulted in more efficient solubilization in liquid refills. However, only 25 to 50% of the initial THC was recovered from the homogenous BHO/liquid refills mixtures containing 3, 5 and 10% (w/w) of decarboxylated BHO (n = 2 at each level). It was hypothesized that the main source of variation was constituted by the partial solubilization of BHO, the formation of an emulsion with many droplets and the absorption of BHO on the walls or bottom of the container. To improve the solubilization of non-decarboxylated BHO in commercial liquid refills, the solution was left for 4 days at 60 °C in a sealed container. Under these soft conditions, partial decarboxylation was observed, leading to a higher percentage of THC (68 ± 4.0%) in BHO diluted to 10% (w/w) in a liquid refill, with a residual content of THC-A (38 ± 12%). However, this soft decarboxylation has rarely been described on Web fora or in the underground literature. To facilitate the solubilization and vaporization of BHO in liquid refills, pure PG was substituted with commercial PG/glycerin mixtures. Pure PG is an alternative to PG/glycerin mixtures also marketed in e-cigarette stores, available for the homemade preparation of liquid refills. Decarboxylated BHO (2 hours at 120 °C) samples were therefore diluted to 3, 5 and 10% (w/w) in pure PG. As reported in Table 2, the solubilization was increased, leading to a THC conversion of 67%, compared to BHO diluted to 10% (w/w) in commercial liquid refills (55%). However, insoluble matter and some fine particulates in the suspension were still noticeable.

PG is less viscous than glycerin and a better emulsifier and solvent. In contrast with glycerin, it is miscible with several apolar solvents. These benefits might explain why a more homogenous and concentrated BHO/PG liquid could be more rapidly achieved. Nevertheless, omission of glycerin greatly reduced the formation of visible white aerosol and could unbalance the organoleptic qualities of the aerosol taste.

Prior heat processing of BHO is strongly recommended to obtain a satisfactory decarboxylation yield of THC-A. However, the combination between the intensity and duration of coil heating cannot be optimal with the standard settings of e-cigarettes. This thermal treatment should therefore be undertaken before BHO mixing with the vaping liquid. Better yields were also observed with soft decarboxylation conditions (i.e., prolonged heating time and medium temperature) applied to BHO/PG mixtures.

#### Experimental BHO “vapability”: cannabinoids concentrations in liquid refills and in vapors of liquid refills (commercial and pure PG)

Table 2 presents the cannabinoids contents measured in liquid refills (commercial and pure PG) fortified with three concentrations of BHO (3, 5 and 10%, w/w) and the cannabinoids contents measured in condensed vapors from these liquid refills.

The quantities of cannabinoids-enriched liquid refills that should be vaped for their therapeutic or alleged beneficial effects were estimated from THC concentrations present in the liquid refill (Table 1) and in condensed vapor (Table 2). To this end, a minimal dose of 1.5 mg of THC was selected, assuming a biodisponibility of 35% for inhaled THC aerosol. Indeed, this quantity has been reported as the lowest effective dose able to induce some psychoactive effects, when injected intravenously^25^,^29^. Similar doses have been reported for therapeutic administration of THC. For instance, oral THC doses from 2.5 to 10 mg daily have been used to treat amyotrophic lateral sclerosis^30^ while 2.5 mg of oral THC taken daily for four weeks after meals was used to increase the appetite of people with cancer^31^. An oromucosal spray (Sativex^®^, containing 2.7 mg of THC and 2.5 mg of CBD) has been used at a dose of 2.5–120 mg in divided doses for up to eight weeks^32^. A low dose of cannabis (1.3% THC, 0.8 g, i.e., approximately 10 mg THC) was vaporized and inhaled in 4 successive puffs using the Volcano^®^ table vaporizer (Storz & Bickel, USA) to improve neuropathic pain significantly^33^. For appropriate comparison, the estimated quantities of liquid refills were expressed in puffs, assuming a consumption of 100 mg of liquid for 20 puffs of 70 mL (the value commonly observed herein).

Assuming a vaporization yield of 100% for the THC contained in the liquids, the inhalation of 12 to 15 puffs of 70 mL of aerosol from liquid refills fortified with decarboxylated BHO titrated at 10% of THC should be sufficient to generate the expected therapeutic effects with minimal undesired psychoactive side effects. BHO concentrations higher than 10% could be used to increase the therapeutic effects and simultaneously to reduce the number of puffs. However, achieving a clear and homogenous solution will be difficult due to flaws in solubility. We can also speculate that some users will experiment with e-cigarettes for recreational reasons. To feel effects similar to those from the smoking of traditional cannabis cigarettes made with the same plant material (400 mg of tobacco, 400 mg of cannabis for 17% of both THC + THC-A), between 10 and 14 puffs of BHO diluted to 10% in pure PG liquid refill should be inhaled. BHO dilutions greater than 10% could be more effective but in pure PG rather than in commercial liquid refills. Indeed, the macroscopic and microscopic observations, as well as the composition of the diluted decarboxylated BHO in pure PG, confirmed this assumption: higher recovery yields of THC were obtained (61 to 73%). Under these conditions, the vaping of 13 puffs of 70 mL of pure PG fortified with decarboxylated BHO at 10% (w/w) could be sufficient to produce some of the desired effects. Similarly, the inhalation of only 11 puffs of 70 mL from this mixture should deliver the same THC quantity as that recovered from the smoke of real cannabis cigarettes (400 mg of tobacco, 400 mg of cannabis at 17% of both THC-A and THC).

However, the amounts of cannabinoids liquid refills listed in Table 2 are likely overestimated because we assumed that the vaporization of the liquid and cannabinoids was conducted with a yield of 100%. Experimental results, obtained from our vaping setup, indicated that much higher amounts of liquid refill would be required to produce the same effects of cannabis as expected with regard to theoretical simulation discussed above (Table 2). The comparison of the cannabinoids concentrations in the parent liquid refill and the consecutive vapor obtained from the BHO-enriched commercial liquid refill revealed that only 5 to 24% (strong decarboxylation) and 6 to 24% (soft decarboxylation) of THC present in the liquid was recovered from the aerosol. Consequently, the choice of the decarboxylation procedure had a limited effect on the yield and concentration of THC found in the aerosol. The substitution of pure PG for the commercial PG/glycerin mixture had a greater impact on the vaporization, resulting in a more concentrated and homogenous liquid, together with a higher level of THC in the aerosol. Using pure PG instead of commercial liquid, the THC content in BHO at 10% in PG was similar to the THC content in softly decarboxylated BHO in commercial liquid, and only 14% of THC present in the liquid was recovered in the aerosol. As a result, the vaporization yield of THC in the selected experimental setup was much lower than expected. An atomization yield of a maximum of 25% seems more realistic.

The quantities of each solution of BHO in commercial liquid refills (3, 5 and 10% w/w, n = 2 for each concentration) and pure PG (10% w/w, n = 3) required to reach minimal therapeutic or recreational effects (equivalent to 1.5 mg of THC, i.v.) under the experimental conditions used (e-cigarette settings and pattern of vaporization) are compiled in Table 3. Under these conditions and assuming that a mean quantity of 100 mg was consumed in 20 puffs of 70 mL, a minimal number of 200 puffs (commercial liquid refill) or 95 puffs (pure PG), both spiked with decarboxylated BHO (10% w/w), could be sufficient. In addition, under the same assumptions as before, a minimal number of 174 puffs (commercial liquid refill) or 83 puffs (pure PG), both spiked with decarboxylated BHO (10% w/w), is thus required to obtain the quantity of THC present in the smoke of a real cannabis cigarette (400 mg of tobacco, 400 mg of cannabis at 17% of both THC-A and THC).

### Carbonyls and VOCs contents

To improve the efficiency of cannabinoids vaporization, i.e., the transformation of cannabinoids from the liquid refill mixtures to the vapor phase, the most extreme e-cigarette heating conditions were selected. These conditions were achieved with the maximal settings combination (highest power and voltage) of the e-cigarette chosen for the study. These settings should increase the decarboxylation rate of acid-cannabinoids but should also simultaneously produce more contaminants. However, if these high settings are not chosen by conventional vapers because they lead simultaneously to “dry puffs” with unpleasant organoleptic qualities, it is important to characterize the device from a chemical and sanitary point of view. Indeed, the strong flavor of the terpenoids compounds of cannabinoids mixtures can mask the bad taste of commercial liquid refills vaped at high settings.

The results concerning carbonyls and VOCs concentrations found in the vapors generated from liquid refill mixtures are compiled in Table 3. Only two carbonyls were detected in significant amounts (formaldehyde and acetaldehyde) and only PG as a VOC.

Assuming a consumption of 100 mg of liquid refill in 20 puffs of 70 mL (mean amount determined during all of the experiments), the formaldehyde content in commercial liquid refills ranged from 0.5 to 22 μg, while the acetaldehyde content was between 0.9 and 18 μg. No relationship between the concentration of nicotine (according to the manufacturer’s specifications) and the quantities of carbonyls could be found.

The carbonyls amounts measured were similar to those previously reported (formaldehyde: 0.2 to 5.6 μg; acetaldehyde: 0.1 to 1.4 μg for 15 puffs of 70 mL)^19^,^34^. Nevertheless, the acetaldehyde concentrations appeared to be higher in our study, probably likely because of the differences in device, settings and liquid refill compositions. Moreover, we could show a direct correlation between the e-cigarette settings and the concentrations of carbonyls: 6.6 W of heating power resulted in a minimal vaporizing coil temperature of 165 °C. Under these conditions, the carbonyls amounts reported herein were less than those reported in other studies with a cumulated carbonyls quantity of 380 μg, generated by 10 successive puffs (voltage: 5 V)^12^.

By the same logic, the formaldehyde amounts (ranging from 4 to 42 μg) in vapors generated from BHO mixtures (dissolved in liquid refills or pure PG) did not significantly differ from those released during the atomization of commercial liquid refills, although a slight increase could be noted. Acetaldehyde amounts (ranging from 10 μg to 110 μg) were correlated with the cannabinoids levels and BHO concentrations. Higher acetaldehyde amounts were obtained after vaping BHO mixtures dissolved in commercial liquid refills or pure PG, rather than after vaping the commercial liquid refill alone.

As expected, PG was detected in the vapors of commercial liquid refills (spiked with or without cannabinoids standards) made of pure PG or of commercial liquid refill composition. The results appeared random because the glycerin level indicated by the manufacturer (30%) could disturb PG trapping by charcoal filters). PG was more easily detected when BHO was dissolved in pure PG (Table 4). Concentrations of PG in the vapors, ranging from 35 to 96 μg/mg of liquid refill, were found during the experiments.

Finally, the VOCs amounts in vapors generated from e-cigarettes were directly compared to those obtained from the smoke of real tobacco cigarettes and regular cannabis/tobacco cigarettes (1:1, w/w). No VOCs were detected in the vapors of the different liquid refills (commercial, cannabinoids standards and BHO mixtures) except for PG, whereas acetone, benzene, toluene, ethylbenzene, p-xylene, styrene and phenolic compounds were present in tobacco cigarettes and real cannabis cigarettes (Table 4). However, the smoke of regular cannabis cigarettes seems to contain less acetone and styrene and more phenolic compounds than that of regular tobacco cigarettes.

## Discussion

### Feasibility and efficiency of cannabis vaping

The combustion and inhalation of cannabis cigarettes is generally considered an inappropriate method for the therapeutic administration of cannabis. Safer and healthier alternatives for consuming cannabis have been proposed to minimize the risks associated with the inhalation of toxic pyrolytic by-products. Vaporization conducted at less than combustion temperatures is one of the best recommended alternative methods to cannabis smoking. A vaporizer heats the cannabis plant material at a moderate temperature, causing the active cannabinoids to evaporate into an aerosol that contains far fewer harmful components. To this end, table vaporizers are commercially available (e.g., Volcano™, Storz & Bickel Gmbh); however, they are not portable, compact or sturdy. Portable, robust, low-weight, pocket pen-vaporizers are now available for nicotine inhalation. Many of these portable vaporizers can be adapted for the consumption of cannabis extracts. E-cigarettes of different brands and of varied designs could also be used for this purpose. To this end, e-cigarettes equipped with various special vaporization chambers can be used to deliver a therapeutic dose of cannabinoids. Roughly three types of devices exist on the market: those able to atomize ground plant material; those made for cannabis wax vaporization; and those intended for the atomization of liquid solutions. Vaporization of ready-to-use, pre-packaged liquids, having a certified composition and concentration, appears to be the most convenient and easiest option for therapeutic use by patients.

In this context, the possibility of delivering cannabinoids with e-cigarettes has led to a new method to administer therapeutic drugs and cannabis-based medicine, which we suggest calling “therapeutic cannavaping”. The manufacture of cannabinoids-enriched e-liquids was found to be quite difficult, and achieving a homogenous and stable mixture of high-potency waxy residues of cannabis extracts (e.g., BHO) with polar and viscous PG/glycerin commercial e-liquids is an ongoing challenge. In our study, BHO concentrations up to 10% (w/w) could be obtained. However, they were not sufficiently concentrated because inhalation of approximately 100 puffs of 70 mL would have been necessary to induce the same effects than those caused by intravenous THC administration of 1.5 mg. Preparation of liquids with higher BHO content could be of significant interest for therapeutic cannabinoids delivery as an alternative to cannabis smoking, especially for practical reasons. However, higher BHO concentrations can lead to cannabinoids solubility problems in the liquid refills. Optimization research, in particular with PG, should be undertaken to determine the best liquid refill composition offering a certified cannabinoids concentrations in the liquid and, if possible, in the vapor. Pure PG and other blends of glycols (e.g., short chain PG liquid mixtures) should be tested for the manufacture of BHO concentrates as e-liquids suitable for vaporizers and e-cigarettes.

Concerning misuse of “cannavaping”, the high number of puffs required to induce minimal psychoactive effects could be considered a rebuttal to “cannavapers” who wish to experience the same effects as real cannabis cigarettes with e-cigarette. To feel the first psychoactive effects sooner, increases in BHO levels and THC concentrations in the vaped solution are required, but due to the inconvenience in its manufacture and final organoleptic properties, such goals will be made more difficult and less appealing. In our opinion and according to other scientists, recreational “cannavaping” remains possible with e-cigarettes; however, its poor efficiency makes the risk of observing a new recreational cannavaping trend unlikely^5^.

Moreover, the most common commercial liquid refills (containing PG and glycerin in various proportions and often some water) are not the best solvents to solubilize BHO. Pure PG is a better solvent. Moreover, it requires the addition of flavorings as in “homemade” liquid refills because the vapors of concentrated BHO in pure PG-based liquids have been reported by users to promote throat hits, defined as pleasant feelings in throat, and to exhibit unpleasant organoleptic properties. Liquid refill choice is important to guaranteeing fair BHO solubilization and THC availability in the vapor, and it requires low glycerin content. However, glycerin is responsible for the “white vapor” noticeable when vaping devices are used, and its presence is advocated by conventional vapers. In contrast, the heating of glycerin can lead to an increase in acrolein, another toxic contaminant (not reported in our experiments because of the liquid refill composition used with no or low glycerin content). Consequently, the preparation of BHO and the making of a homogenous homemade liquid refills containing high proportions of BHO, with a pleasant taste and a fair level of glycerin, appear complex and unappealing to recreational and addicted cannabis consumers.

Thus, in our opinion, therapeutic “cannavaping” might be the most interesting outcome for the cannabinoids use with e-cigarettes. This gentle method of THC delivery could be of great importance in the context of finding new administration methods for medical cannabis. Indeed, “cannavaping” avoids the inhaling of significant amounts of toxic contaminants released during the combustion of regular cannabis cigarettes, and it guarantees soft and reproducible THC delivery if the spiked liquid refill composition is controlled. In this context, “cannavaping” could alleviate some symptoms that have been previously treated with some success with medicinal cannabis using other routes and methods of administration. “Cannavaping” could also be recommended to patients who want to quit cannabis smoking or to reduce significantly the importance and frequency of their consumption and possibly attenuate withdrawal symptoms^35^.

However, because the manual settings of more modern e-cigarettes can be freely adjusted by the vaper, unsafe settings can be selected to increase the efficiency of BHO and THC vaporization. It is therefore important to monitor carbonyls and VOCs contaminants to keep these potentially toxic residues in negligible quantities. Moreover, the “cannavaping” of BHO diluted in liquid refills can also lead to the formation of specific toxic contaminants. If the vaping of these liquid refills at these high settings produced unpleasant flavors due to liquid burning, these off-flavors could be masked by the fragrance of cannabis terpenoids. The results compiled in this study showed that the aerosols produced by the vaporization of BHO mixtures (up to 10%, w/w) at the highest e-cigarette settings (6.4 W, 5 V) did not generate new specific contaminants. However, these high settings were correlated with the formation of formaldehyde and acetaldehyde content in not negligible but similar amounts to those already determined with commercial liquid refills and e-cigarettes. Therefore, we advocate testing for the presence of toxic contaminants when optimizing the vaporization of cannabinoids-enriched liquid refills. In particular, we recommend determining the levels of carbonyls and VOCs contaminants, although the first results obtained at high settings did not seem to show abnormalities.

### Limitations

The maximal levels of power and voltage delivered by the e-cigarette were chosen to maximize the contaminants generation and the recovery of cannabinoids in the vapor. However, these settings could be ignored by vapers because of organoleptic flaws.

We used cannabinoid standards and BHO, a viscous, amber-colored, waxy cannabis concentrate, as raw cannabinoids material, but other sources, such as tinctures or macerated preparations, could be used by vapers also, although irritating ethanolic vapors could deter some users. This study specifically focused on homemade cannabinoids enriched liquid refills with BHO, but illegal liquid solutions of synthetic cannabinoids are currently available on the Internet^6^,^36^.

The most dangerous aspect of BHO concerns its preparation. Several reports of gas explosions while attempting butane extraction have been posted on the Internet. Use of a fume hood is mandatory in laboratory settings. Inhalation of traces of butane appears to pose no major health risk, although death reports correlated with butane inhalation have concerned the abuse of gas fuel among drug addicts^37^,^38^. Supercritical CO_2_ extraction is very likely more appropriate for preparing cannabis concentrates. CO_2_ toxicity is lower, and the risk of explosion is far less. However, the preparation of “CO_2_HO” concentrate requires more sophisticated and costlier equipment.

In the calculations, bioavailability of vaped and smoked THC is supposed to be the same. However, measurement of cannabinoids compounds in the aerosol of e-cigarettes and smoke of cannabis cigarettes does not necessarily imply equivalent absorption to the bloodstream. The different vehicles of transport of cannabis compounds could result in differences in the speed and rate of absorption from the respiratory tract. This can be assessed only by measuring plasma levels.

A limitation of the setup for the temperature measurements is that the measuring site inside the wick is not similar to the temperature on the surface of the wick where the evaporation takes place. Differences between different measuring sites may be occur, with the temperature becoming lower as you move away from the center of the coil.

Only one common type of e-cigarette was assessed for THC delivery in this study. However, other portable devices and brands with more or less sophisticated designs are currently sold and marketed. They can produce similar, but also different cannabinoids, carbonyls and VOCs amounts. Higher levels of impurities could be formed, especially with vaporizers delivering high voltages or having larger energy supplies, and a high puff frequency could lead to coil overheating and contaminant generation. Similarly, only one type of clearomizer has been tested, while many other models of e-cigarettes (with cartomizers or clearomizer/atomizers, single or dual coils, with or without wicks, for example) can be found in specialized shops and on the Internet. Their use with cannabinoids could also produce different cannabinoids, carbonyls and COVs amounts. The toxicity of the vapors generated by e-cigarettes has already been demonstrated in several studies^39^. However, extrapolation of these results to other models of e-devices and liquid refills is questionable.

## Conclusion

Cannavaping appears to be a gentle, efficient, user-friendly and safe alternative method for cannabis smoking for medical cannabis delivery. Its expected benefits very likely overcome the advantages of oral administration because ingestion is characterized by its erratic absorption and poor biodisponibility. Ingested compounds that undergo first pass metabolism could be less active than inhaled compounds, which would have direct access to the bloodstream without being metabolized first. Moreover, cannavaping could avoid emesis due to strong doses of therapeutic cannabinoids when smoked or inhaled through vaporization. However, potential misuse of cannavaping has been identified, based on dabbing practices and widespread accessibility to more sophisticated e-cigarette devices. BHO can be easily extracted at home, and electronic devices specifically designed for dabbing or vaping are available on the market, as well as edible solvents to produce homemade liquid refills enriched with cannabinoids. Consequently, recreational or addictive cannavaping is theoretically possible. However, the poor solubility of BHO in commercial liquid refills (especially those with high glycerin content) prevents achieving high BHO concentrations, which are very likely preferred by recreational cannavapers and dabbing consumers. Illegal cannavaping is suspected to present a low risk of becoming popular among cannabis smokers. Public health actors and stakeholders must pay attention to this potential misuse, but safety surveys and police work should be more focused on cannabis dabbing than cannavaping. Therefore, the likelihood of misuse of cannavaping seems to be very limited, whereas therapeutic applications of cannavaping have undeniable benefits over other administration routes, with the controlled dosage of cannabinoids-enriched liquid refills. Similarly, the electronic devices commercialized for therapeutic cannavaping should be carefully studied to prevent potential overheating and contaminants generation.

## Materials and Methods

### Cannabinoid standard, liquid refills and e-cigarettes

Cannabinoids standards were purchased from THC Pharm (Frankfurt, Germany) (THC-A, 1 mg/mL methanol; Cannabidiol-A [CBD-A], 1 mg/mL; cannabigerol, 1 mg/mL methanol) and from Lipomed (Arlesheim, Switzerland) (THC, 1 mg/mL methanol; cannabinol [CBN], 1 mg/mL methanol; cannabidiol [CBD], 1 mg/mL methanol). The acetonitrile from Sigma-Aldrich (Steinheim, Germany), methanol from Merck (Darmstadt, Germany), and ethanol and formic acid from Fluka (Buchs, Switzerland) were all of analytical grade.

Three e-cigarettes (iTaste VV type, VV standing for variable voltage from 3.5 to 5 V), clearomizers (CE4+ type, 1.6 mL, 4 long wicks) and liquid refills (DEA Calliope™, Dea Flavour S.R.L., Trento, Italy), with different nicotine contents (nicotine-free, 9 mg/mL and 18 mg/mL) were purchased from local specialized e-cigarette retailers in Lausanne and Yverdon-les-Bains (Switzerland). These e-cigarettes (coil resistance of 3.8 Ω) have been chosen because of their different setting options (power and voltage). For the experiments, the voltage was set at the maximum permitted level (5 V). The composition of the liquid refills was the same for except the nicotine burden: propylene glycol (PG) 60%, vegetal glycerin (VG) 30% and flavors (+nicotine if present) in 10% distilled water.

### Preparation of cannabinoid-enriched liquid refills

To study the efficiency of decarboxylation by e-cigarettes, standards of THC-A (50 mg of pure powder) and THC (in ethanolic solution at 100 mg/ml), obtained from Lipomed (Arlesheim, Switzerland), were mixed with liquid refills.

**Figure 2:**
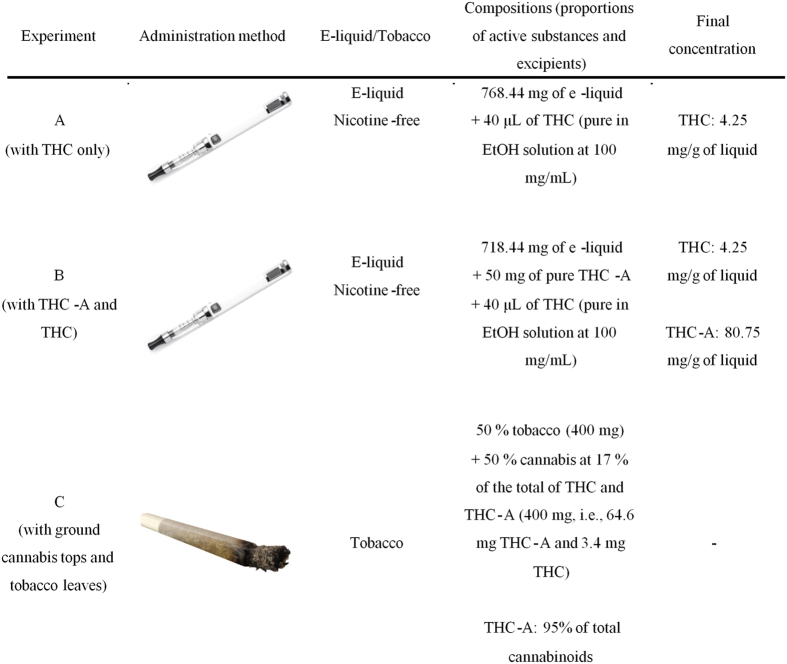
Decarboxylation experimental protocols. (1 g of liquid is consumed after about 200 puffs and the described cannabis cigarette is consumed after 15–20 puffs).

Many users on the Internet have reported performing a decarboxylation step to convert THC-A into its psychoactive THC form. Commercial nicotine-free liquid refills (n = 2) were spiked with pure THC ethanolic solution up to a concentration of 4.3 mg/g of liquid (Design A). Another set of commercial nicotine-free liquid refills (n = 2) were spiked with pure THC ethanolic solution up to a concentration of 4.3 mg/g of liquid and pure THC-A powder up to a concentration of 81 mg/g of liquid (Design B). Finally, a typical cannabis cigarette (“joint”) was prepared according the European practice (Design C): chopped head tops mixed with tobacco (400 mg of tobacco, 400 mg of cannabis at 17% of a total of THC + THC-A, composed of 95% THC-A) were used to generate theoretical cannabinoids quantities similar to those used in the designs A and B. The cannabis used was medical grade cannabis (bedrobinol from Bedrocan BV, Veendam, the Netherlands) with 17% of a total of THC + THC-A and less than 1.0% CBD. The three designs are compiled in Fig. 2.

To evaluate the efficiency of the decarboxylation step for BHO before vaping, known amounts of decarboxylated (thermally activated) and not decarboxylated (not activated) BHO were dissolved in commercial liquid refills and pure propylene glycol (PG) purchased from Chemnovatic (Lublin, Poland). BHO at 3%, 5% and 10% (w/w) in nicotine-free e-liquid and pure PG was prepared.

### BHO preparation

**Figure 3:**
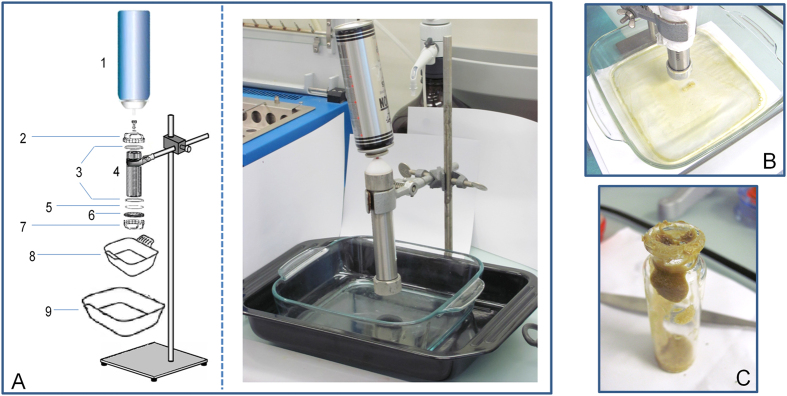
BHO fabrication design. ((**A**) Extraction setup [1. Butane lighter refill, 2. Teflon screw with central hole for can nozzle, 3. Rubber joints, 4. Cylindrical steel extractor, 5. Paper filter, 6. Wire sieve, 7. Steel screw with large central hole, 8. Pyrex recipient for BHO collection, 9. Plate for boiling water], (**B**) BHO in the plate after butane evaporation, (**C**) BHO paste collection).

A known quantity of Bedrobinol (used in Design C) was placed in the metallic extractor, and BHO was prepared following a home-made protocol adapted from a published patent^40^ and from Web fora^8^ (Fig. 3).

Briefly described, the experimental protocol included four major steps: first, introduction of ground head tops in the steel extractor; second, butane extraction under the hood; third, scraping of the waxy residue; and fourth, decarboxylation of the residue in the oven. No further purification (winterization) was performed. The protocol was deliberately not more detailed here to avoid malicious duplication. The detailed protocol remains available upon request for scientists.

### Composition of liquefied butane gas

The extraction gas was analyzed following published procedures^41^,^42^. The composition of butane gas cans used to extract BHO was calculated by comparison with data obtained with two certified propane/isobutane/butane gaseous mixtures (GTS Sapray, Gruppo Autogas Nord, Genoa, Italy). Their chemical compositions were as follows (main components in approx. weight):

-first brand: propane 25%, isobutane 20% and butane 55% (in weight); and

-second brand: propane 62%, isobutane 14% and butane 24% (in weight).

Although the packaging stated, “almost zero impurities”, the gas used to extract BHO was a mixture of propane 21%, isobutane 69%, and butane 10% (in weight). As a result, the cannabinoids concentrates were extracted mainly by isobutane and should be called iBHO for iso-butane hashish oil.

### Quality control measurement of prepared BHO and liquid refill

Cannabinoids in BHO extracts and in spiked liquid refills with BHO were quantified by HPLC using Agilent 1100 Series equipment with diode array detection. The cannabinoids were separated by gradient elution on a Macherey-Nagel (Düren, Germany) CC 250/3 Nucleodur 100–5 C8 ec column, protected by a MN CC 8/3 Nucleodur 100–5 C8 ec precolumn. Reversed phase gradient elution was obtained by mixing two eluents: A) methanol/water (1:1, v/v) and 25 mM formic acid; and B) methanol and 25 mM formic acid. The column flow was 0.5 mL/min, and the runtime was 30 min. The initial proportion of solvent B was 40%. This proportion was linearly increased up to 100% in 25 min. The final eluent composition was maintained for 3 min. A linear calibration curve was obtained between 5 and 100 μg/mL with THC-A, THC, CBN, CBD and CBD-A. Cannabinoids were identified by comparing their retention time and UV spectrum with those of authentic samples and by performing a library search in the Pragst, Herzler, Herre, Erxleben, and Rothe 2001/2007 UV-Vis databases. The library was completed with the spectra of all commercially available cannabinoids. UV spectra were registered between 195 and 300 nm with a range step of 2 nm. Samples were diluted to an appropriate cannabinoids concentration ranging between 20 and 100 μg/mL. Ten microliters of diluted extract in solvent A were injected onto the column.

### Vaping device

**Figure 4:**
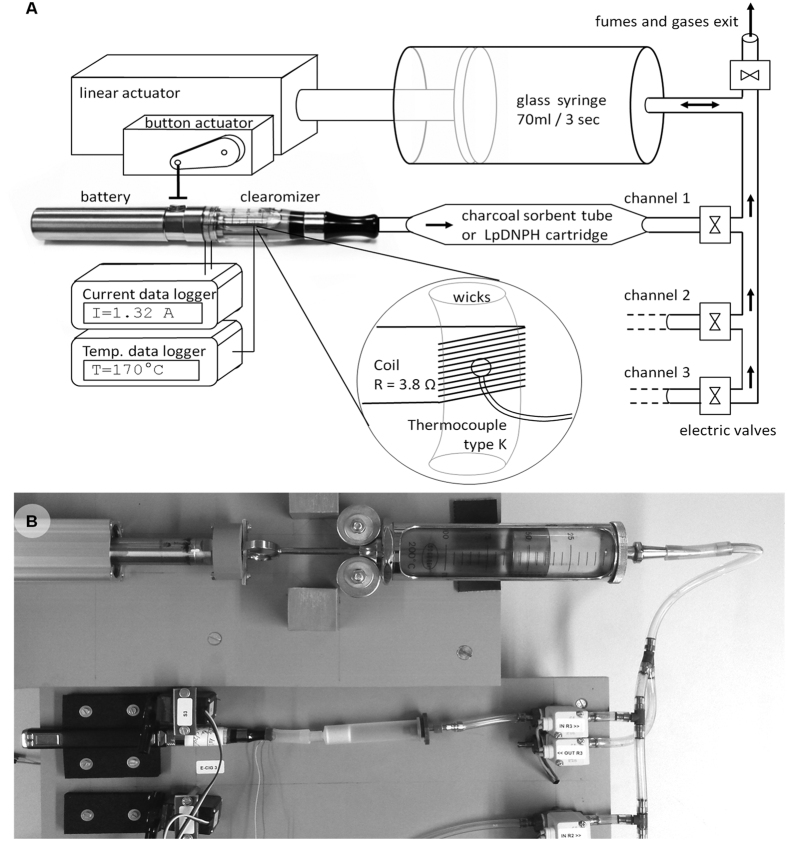
Operating diagram and picture of the vaping device used to sample the gases and aerosols generated by the e-cigarettes. ((**A**) Diagram of the main parts of the vaping device with a focus on the temperature sensor location, (**B**) Photograph of the linear actuator, the glass syringe, and the first e-cigarette channel).

Aerosols from e-cigarettes were generated using a smoking/vaping device specifically conceived and designed for the study (Institute for Work and Health, Lausanne, Switzerland) (Fig. 4). It is a three-channel linear piston-like smoking machine with adjustable puffing frequencies and volumes, controlled by a computer interface. Cartridges and special traps were placed between the clearomizer and the suction syringe-pump to trap volatile organic compounds (VOCs), carbonyls and cannabinoids in the generated aerosol. In a first step, the syringe pump draws aerosol through the clearomizer and then through the sampling cartridge. In a second step, the aerosol moves toward an outlet pipe. The sampling cartridge is located close to the mouthpiece (<5 cm) to limit the loss of aerosols by impaction or condensation inside the tubing. In this study, the smoking machine was set to generate 70 mL puffs with a frequency of 3 puffs per minute^19^. To prevent overheating of the clearomizer caused by too frequent ignition, three e-cigarettes were used alternately in one minute. The puff duration, defined as the time during which the e-cigarette push button is pressed, was 3 seconds. Given that the puff duration is an essential parameter that determines the aerosol temperature, the e-cigarette button was pressed by an electric actuator to ensure precise timing control. The temperature of the e-liquid near the coil was monitored at a sampling rate of 3 Hz (type k thermocouple and MAX31855 MAXIM data logger). The temperature sensor was located inside the wick fibers, near the center of the coil without direct contact with the coil. The electric current through the coil was monitored by a current sensor (INA169 Analog DC Current Sensor) connected between the battery and the coil, at a sampling rate of 3 Hz.

### Volatile organic compounds (VOCs) analysis

To sample VOCs, charcoal sorbent tubes (SKC Anasorb CSC 400/200) were inserted between the e-cigarette mouthpiece and the corresponding electric valve (see Fig. 4). The tubes were removed after 20 cycles of 70 mL puffs at 3 seconds per puff, with a frequency of 2 puffs per minute. After collection, the front and the back of each tube were desorbed in 5 ml of CS_2_ (Sigma Aldrich, Buchs, Switzerland).

All of the calibration points were prepared by spiking the front of the charcoal sorbent tube. In this study, only the front was analyzed, assuming that none of the tube was saturated because all of the sample contents were always below the highest calibration point of the measured compound.

A first screening of VOCs was performed by gas chromatography (HP Agilent 6890) coupled to a mass spectrometer (HP Agilent 5973). Compounds identified with mass spectra libraries were quantified by gas chromatography coupled to a flame ionization detector (GC-FID) (Varian 3800, column CP-SIL 8CB 60 m, 0.25 mm i.d., 0.25 μm film thickness), split set to 20%, an injection temperature of 200 °C and an oven temperature gradient of 50 °C to 165 °C. Five standards were prepared from 200 to 900 μg/tube for each compound. Due to the high expected amount of VOCs and to prevent saturation of the solvent, tobacco and cannabis samples were desorbed in 10 ml of CS_2_.

Phenols and propylene glycols were analyzed by GC-FID (Agilent 6890, column Supelcowax 30 m, 0.25 mm i.d., 0.25 μm film thickness), split set to 20%, with an injection temperature of 250 °C and an oven temperature gradient of 160 °C to 200 °C. Five standards were prepared from 0.2 to 1.1 mg/tube for phenols and 0.2 to 25 mg/tube for propylene glycol.

### Carbonyls analysis

Regarding VOCs sampling, carbonyls were trapped using LpDNPH S10 cartridges (Supelco, 350 mg, 3 mL) coated with 2,4-dinitrophenylhydrazine (2,4-DNPH). For tobacco and cannabis cigarettes, the LpDNPH H30 cartridges (Supelco, 1 g, 6 mL) were used to avoid overloading the cartridge carbonyl capacity. To sample, cartridges were also inserted between the e-cigarette mouthpiece and the corresponding electric valve (Fig. 4). To quantify carbonyls, the method was based on the reaction of carbonyls with 2,4-DNPH to form the corresponding hydrazone derivative. After sampling, the cartridges were desorbed using 3 ml of acetonitrile. Eluate was filtered using syringe filters with PTFE membrane (Acrodisc CR 13 mm PN 4422T HPLC certified), and the volume was adjusted to 3 ml with acetonitrile in a 4 ml glass vial. Five calibration points were prepared in acetonitrile at concentrations ranging from 1.6 to 53 ng/μl. Samples were analyzed by HPLC (Varian ProStar Model 410) connected to a UV detector (Varian ProStar Model 335). Carbonyls were separating using a Spherisorb ODS 1 column (150 × 4.6 mm, 3 μm film thickness) at a rate of 0.8 ml/min, and kept at 30 °C. The gradient elution was obtained by mixing two eluents: A) methanol/water (5:5, v/v); and B) methanol. The initial proportion of solvent A was 100% for 3 minutes; then, the proportion of solvent B linearly increased up to 100% in 27 min and was maintained for 5 min. The injected volume was 10 μl, and the UV spectra were set at 365 nm.

### Cannabinoids analysis

Quantitative analysis was performed by liquid chromatography coupled to a tandem mass spectrometer (LC-MS/MS) with selected reaction monitoring (SRM) mode. The UltiMate 3000 LC system from Dionex, coupled to a triple quadrupole API 5000 MS system from AB Sciex, was used. The LC separation was conducted on a Kinetex C18 (Phenomenex), 150 × 2.1 mm (i.d.) column with ammonium formate 5 mM pH 6.8 and acetonitrile as the mobile phase at a flow rate of 0.4 mL/min in gradient elution mode. The SRM transitions used for the quantification of THC-A and THC were *m/z* 357.0 > 313.0; 357.0 > 245.0 and *m/z* 315.2 > 193.0; 315.0 > 259.0, respectively. The method was fully validated according to international guidelines^43^.

## Additional Information

**How to cite this article**: Varlet, V. *et al*. Drug vaping applied to cannabis: Is “Cannavaping” a therapeutic alternative to marijuana? *Sci. Rep.* **6**, 25599; doi: 10.1038/srep25599 (2016).

## Figures and Tables

**Table 1:** Concentrations of cannabinoids (THC, THC-A and both THC + THC-A) in BHO-diluted mixtures in nicotine-free commercial liquid refills.

[% w/w] in liquid refill	Soft decarboxylation (4 days, 60 °C) BHO in commercial e-liquid						Strong decarboxylation (2 hrs, 120 °C) BHO in commercial e-liquid						Strong decarboxylation (2 hrs, 120 °C) BHO in pure PG						Smoke of real joint (400 mg tobacco, 400 mg herb at 17% of sum of THC + THC-A)
	BHO 3%		BHO 5%		BHO 10%		BHO 3%		BHO 5%		BHO 10%		BHO 3%		BHO 5%		BHO 10%		
[%] THC	0.9	1.1	1.4	1.0	7.1	6.5	1.4	1.4	1.4	1.4	5.3	5.6	1.9	2.2	3.1	3.8	6.1	7.3	3.75 mg
[%] THC-A	0.3	0.3	0.7	0.5	4.6	2.9	_	_	_	_	_	_	_	_	_	_	_	_	9.4 μg
[%] Sum of THC + THC-A	1.2	1.4	2.1	1.5	11.7*	9.4	1.4	1.4	1.4	1.4	5.3	5.6	1.9	2.2	3.1	3.8	6.1	7.3	3.75 mg
Quantity of cannabinoid liquid refill required to produce similar effects to 1.5 mg of THC i.v. (mg)**	476	390	306	429	60	66	306	306	306	306	81	77	226	195	138	113	70	59	
Equivalent in puffs of 70 mL	95	78	61	86	12	13	61	61	61	61	16	15	45	39	28	23	14	12	
Quantity of cannabinoid liquid refill required to induce effects similar to THC contained in the smoke of a real cannabis cigarette (mg)	417	341	268	375	53	58	268	268	268	268	71	67	197	170	121	99	61	51	
Equivalent in puffs of 70 mL	83	68	54	75	11	12	54	54	54	54	14	13	39	34	24	20	12	10	

(Influence of the decarboxylation conditions, estimation of the quantity of liquid refill consumption to reach a THC concentration of 1.5 mg i.v. [minimal concentration reported for pharmacological effects] and THC and THC-A amounts measured in the whole smoke of a real cannabis cigarette [400 mg tobacco, 400 mg dronabinol cannabis flower tops containing 17% of both of THC + THC-A]).

*Total cannabinoids amount is higher than BHO percentage = BHO concentrate is not homogenous.

**Assuming a bioavailability of 35% for the cannabinoids contained in the vapor^25^,^29^. Bioavailability of vaped and smoked THC is supposed to be the same.

**Table 2:** Concentrations of cannabinoids (THC, THC-A and both THC + THC-A) in mixtures and vapors of BHO diluted in commercial liquid and pure PG.

[% w/w] in liquid refill	Strong decarboxylation (2 hrs, 120 °C) BHO in commercial e-liquid			Soft decarboxylation (4 days, 65 °C) BHO in commercial e-liquid			Strong decarboxylation (2 hrs, 120 °C) BHO in pure PG			Pure BHO not decarboxylated	Smoke of real joint (400 mg tobacco, 400 mg herb at 17% of [THC-A + THC])	Smoke of real cigarette (800 mg tobacco)
	BHO 3%	BHO 5%	BHO 10%	BHO 3%	BHO 5%	BHO 10%	BHO 3%	BHO 5%	BHO 10%			
THC in liquid [μg/mg of liquid]	14	14	55	10	12	68	69	68	67	2.5		
THC-A in liquid [μg/mg of liquid]	_	_	_	3	6	37.5	_	_	_	765		
THC in vapor [μg/mg of liquid]	3.4	2.3	2.5	0.9	2.9	4.3	Not measured	Not measured	9.0		3.75	0 mg
THC-A in vapor [μg/mg of liquid or /cigarette]	_	_	_	_	0.001	0.004	Not measured	Not measured	_		9.4	0 μg
[THC-A + THC] in vapor [μg/mg of liquid]	3.4	2.3	2.5	0.9	2.9	4.4	Not measured	Not measured	9.0		3.75	0 mg
Quantity of cannabinoid liquid refill required to produce similar effects to 1.5 mg of THC i.v. (mg)	1261	1863	1714	4762	1478	997			476			
Equivalent in puffs of 70 mL	252	373	343	952	296	199			95			
Quantity of cannabinoid liquid refill to induce effects similar to THC contained in the smoke of a real cannabis cigarette (mg)	1103	1630	1500	4167	1293	872			417			
Equivalent in puffs of 70 mL	221	326	300	833	259	174			83			

(Influence of the decarboxylation conditions, estimation of the quantity of liquid refill consumption to reach a THC concentration of 1.5 mg i.v. [minimal concentration reported for pharmacological effects] and THC and THC-A amounts measured in the whole smoke of a real cannabis cigarette [400 mg tobacco, 400 mg dronabinol cannabis flower tops containing 17% of both THC + THC-A]).

*Assuming a bioavailability of 35% for the cannabinoids contained in the vapor^25^,^29^. Bioavailability of vaped and smoked THC is supposed to be the same.

**Table 3:** Carbonyls and volatile organic compounds (VOCs) concentrations measured in the vapors of liquid refills mixtures (commercial, cannabinoids standards and BHO dilutions).

	*n*	Aldehydes		VOCs
		Formaldehyde	Acetaldehyde	Propylene glycol
		*Mean*(*μg/mg e-liquid*)		
E-liquid without nicotine	3	0.14	0.03	150
E-liquid, 9 mg nicotine	3	0.22	0.18	
E-liquid, 18 mg nicotine	3	0.17	0.05	
THC in e-liquid without nicotine	1	0.01	0.01	
THC-A in e-liquid without nicotine	1	0.02	0.02	
BHO 5% (decarboxylated)	1	0.04	0.02	35
BHO 10% (decarboxylated)	1	0.42	0.15	43
BHO 5% (non-decarboxylated) in e-liquid without nicotine	1	0.21	0.46	45
BHO 10% (non-decarboxylated) in e-liquid without nicotine	1	0.29	0.31	96
BHO 5% (non-decarboxylated) in pure PG	2	0.15	0.32	75
BHO 10% (non-decarboxylated) in pure PG	5	0.31	1.1	94

**Table 4:** Carbonyls and volatile organic compounds (VOCs) composition and concentrations determined in the vapors of tobacco cigarettes and regular cannabis cigarettes*.

	Acetone	Benzene	Toluene	Ethyl benzene	p-xylene	Styrene	Phenols
	μg/cigarette*						
Tobacco cigarette(n = 1)	1.05	0.16	0.36	0.07	0.18	1.05	0.75
Dronabinol + tobacco cigarette(n = 1)	0.61	0.16	0.35	0.06	0.12	0.61	0.83

^*^Cigarettes contained 800 mg of tobacco or 400 mg of tobacco and 400 mg of dronabinol (17% of both THC + THC-A).
